# “Freshwater Killer Whales”: Beaching Behavior of an Alien Fish to Hunt Land Birds

**DOI:** 10.1371/journal.pone.0050840

**Published:** 2012-12-05

**Authors:** Julien Cucherousset, Stéphanie Boulêtreau, Frédéric Azémar, Arthur Compin, Mathieu Guillaume, Frédéric Santoul

**Affiliations:** 1 CNRS, Université Paul Sabatier, ENFA, UMR 5174 EDB (Laboratoire Évolution & Diversité Biologique), Toulouse, France; 2 Université de Toulouse, UMR 5174 EDB, Toulouse, France; 3 Université de Toulouse, INP, UPS, EcoLab (Laboratoire d’Ecologie Fonctionnelle et Environnement), Toulouse, France; 4 CNRS, EcoLab, Toulouse, France; Biodiversity Insitute of Ontario - University of Guelph, Canada

## Abstract

The behavioral strategies developed by predators to capture and kill their prey are fascinating, notably for predators that forage for prey at, or beyond, the boundaries of their ecosystem. We report here the occurrence of a beaching behavior used by an alien and large-bodied freshwater predatory fish (*Silurus glanis*) to capture birds on land (i.e. pigeons, *Columbia livia*). Among a total of 45 beaching behaviors observed and filmed, 28% were successful in bird capture. Stable isotope analyses (δ^13^C and δ^15^N) of predators and their putative prey revealed a highly variable dietary contribution of land birds among individuals. Since this extreme behavior has not been reported in the native range of the species, our results suggest that some individuals in introduced predator populations may adapt their behavior to forage on novel prey in new environments, leading to behavioral and trophic specialization to actively cross the water-land interface.

## Introduction

To capture, handle, kill and/or swallow their prey, predators have developed numerous strategies, including pack hunting, venin, cooperation, trapping webs and the use of tools. For instance, savannah chimpanzees have been reported to construct wooden spear-like tools to hunt their preys [1] while New Caledonian crows can use stick tools to capture highly energetic wood-boring beetle larvae [2]. However, perhaps the most astonishing strategies are when the preys targeted by the predator are located outside of the predator’s ecosystem boundaries. These strategies can be grouped into three broad categories. First, the predators can passively lie in ambush until the preys cross the water-land interface (voluntarily or accidently) and capture them. This is notably the case of crocodiles that capture migrating wildebeest crossing rivers and of terrestrial predators such as birds, spiders and bats that capture emerging aquatic insects [3], [4]. Second, some predators may develop strategies that force the prey to enter their ecosystem. For instance, archerfish (*Toxotes jaculatrix* Pallas) have developed complex optical and morphological adaptations to ‘shoot down’ insects located on trees by expelling droplets on the insects that will subsequently fall in the water [5]. Third, and most spectacularly, predators can actively cross the water-land interface to capture the prey. Some marine predators such as killer whales (*Orcinus orca* L.) and bottlenose dolphins (*Tursiops sp.*) display intentional ‘beaching’ behavior to catch prey on beaches [6], [7]. In many predators, these extreme hunting behaviors represent a form of ecological specialization [8], [9] that is displayed only by a subset of individuals in the populations [7]. Here, we report the occurrence of a hunting behavior, analogous to the intentional beaching of marine mammals, in an alien freshwater fish species (European catfish *Silurus glanis* L., the world’s third largest and Europe’s largest freshwater fish) [10], [11] to capture land birds. Additionally, we demonstrate the existence of trophic niche variability within the population with only some individuals foraging on land birds.

## Materials and Methods

### (a) Behavioral Monitoring

**Figure 1:**
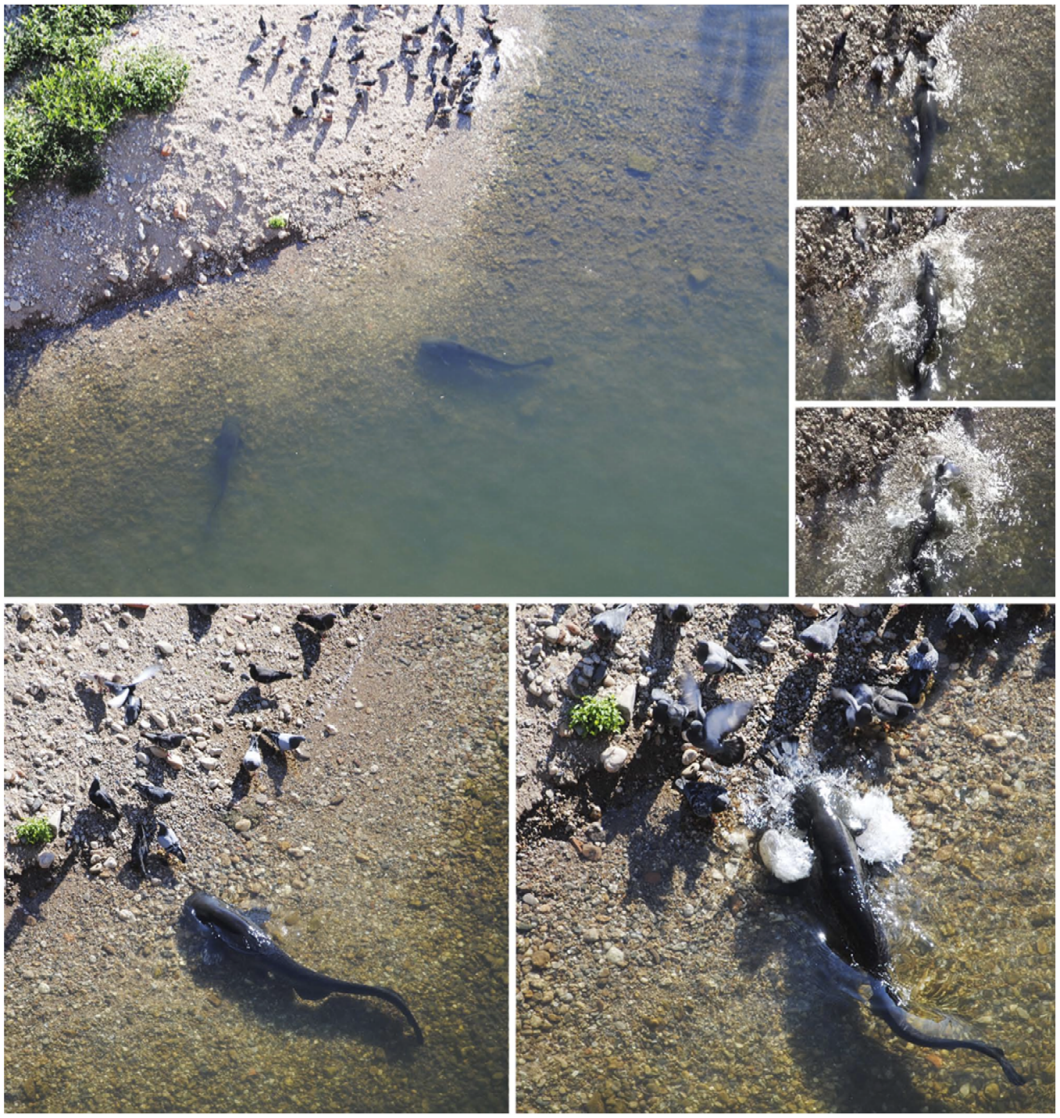
European catfish displaying beaching behavior to capture land birds. Several individuals were observed swimming nearby the gravel beach in shallow waters where pigeons regroup for drinking and cleaning (large picture). One individual is seen approaching land birds and beaching to successfully capture one (small pictures).

European catfish originates from Europe (east of Rhine River) and has been introduced in many ecosystems of Western Europe, including Spain, Italy and Southwestern France [11]. We conducted the present study in the Tarn River (Southwestern France) within the historical city center of Albi, a UNESCO World Heritage Centre. European catfish were introduced in the Tarn River in 1983 and have since established self-sustained populations [12]. Behavioral monitoring was performed from a bridge above a gravel island where pigeons (*Columbia livia* Gmelin) regroup for drinking and cleaning (43° 55′ 51.77″ N, 2° 08′ 41.83″ E). At the studied stretch, the Tarn River is approximately 100 m wide with a mean depth of 3 m (maximum depth 5.4 m) and belongs to a protected area where angling is prohibited. In total, 24 surveys (approx. 3-hour long on average, total observation and filming time of approx. 72 h) were performed from June 30^th^ to October 19^th^ 2011 in the morning or in the afternoon. The number and success rate of beaching behavior were determined by filming the predatory fish nearby the gravel island. Throughout the survey, river discharge was low and the water was clear, allowing full observation of all displayed behavior (Figure 1 and Movie S1).

**Figure 2:**
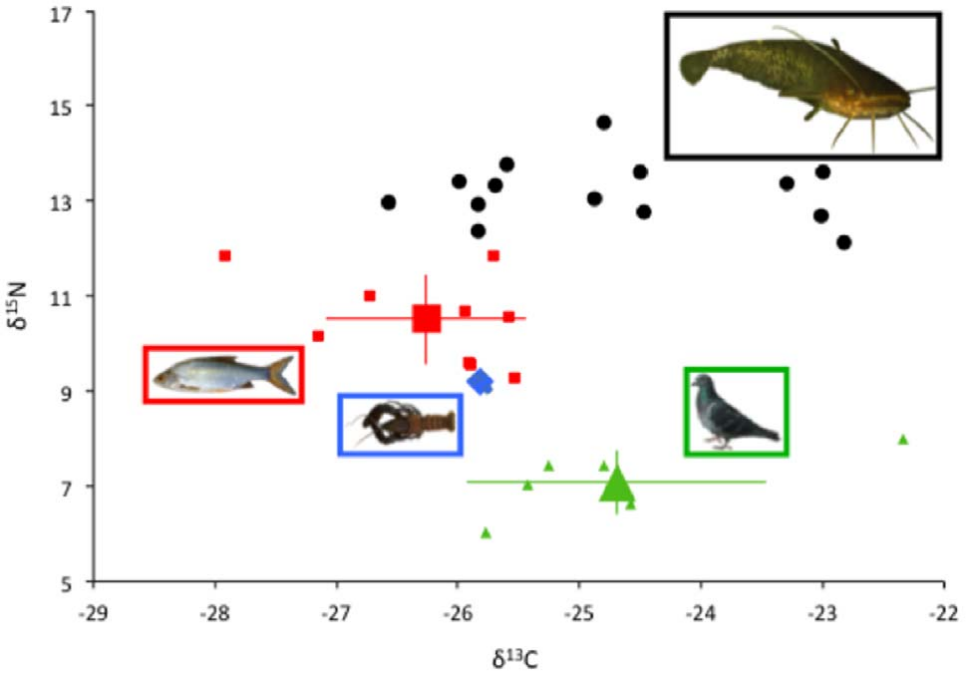
Stable isotope values of European catfish and the putative prey. δ^13^C and δ^15^N values (‰) of each individual (n = 14) and the putative aquatic (fish, n = 9 and crayfish, n = 3) and terrestrial (pigeon, n = 6) prey are displayed. The large symbols for each prey represent the mean value (± SD).

**Figure 3:**
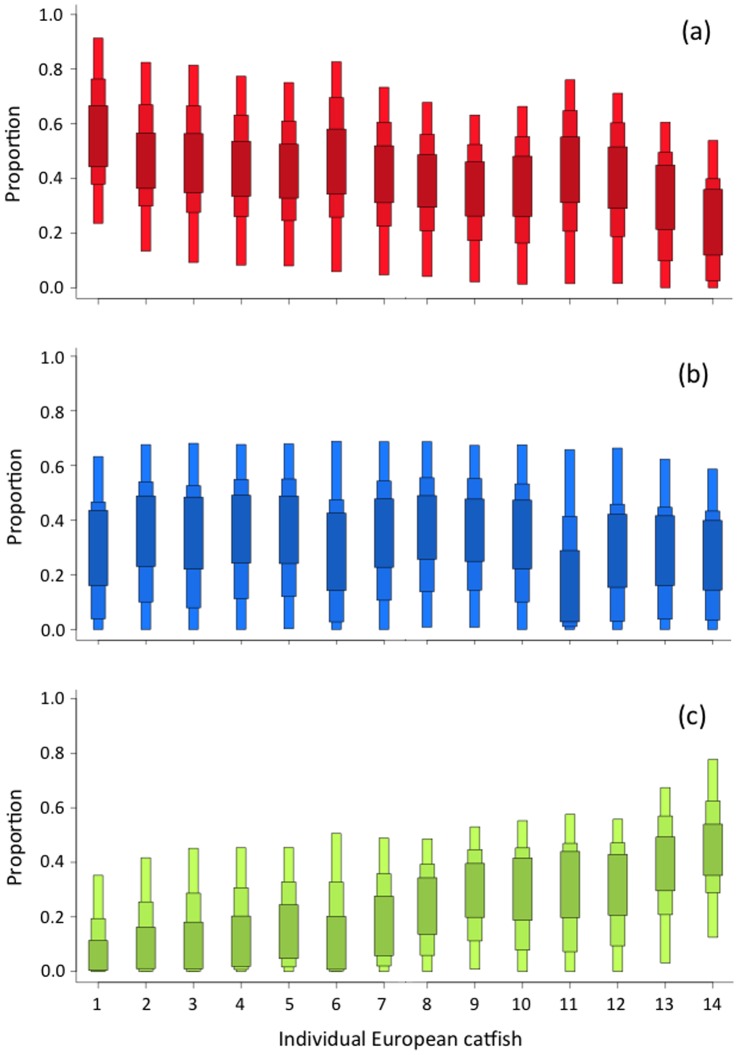
Predicted contribution of putative prey to the diet of each European catfish. Putative prey are (a) fish (in red), (b) crayfish (in blue) and (c) pigeons (in green). Reported values are the lower and upper 50, 75 and 95% Bayesian credibility intervals predicted by the mixing models.

### (b) Stable Isotope Analyses

Tissue samples of European catfish and their potential prey were collected in the observation site from 18^th^ September to 14^th^ October 2011 to quantify the contribution of land birds to the catfish diet using stable isotope analyses. Sampling was allowed by the permit “Arrêté Préfectoral no. 2011–018”. Stable isotope analyses (principally δ^13^C and δ^15^N) are now commonly used by ecologists to provide reliable estimates of long-term dietary patterns of predators [13], [14], including trophic specialization [15], [16] and the ecological impacts of non-native fish species [14], [17]. A total of 14 European catfish located within the study area (estimated body size ranging from 900 to 2000 mm) were sampled. Fin clips were collected by angling from 5 individuals while muscle samples were collected on the flank of 9 additional individuals by scuba divers using a spear gun equipped with a 40-mm length and 27-mm diameter stainless steel biopsy tip. Fin and muscle samples were pooled in the analyses since their stable isotope values do not differ significantly in this species [12]. No lipid correction was performed since samples have low and homogeneous C:N ratios (3.2±0.2 SD) [18]. Muscle samples of the putative aquatic and terrestrial prey present in the study area were also collected. These included crayfish (*Orconectes limosus* Rafinesque n = 3) and Cyprinids prey fish (*Blicca bjoerkna* L. n = 3, *Cyprinus carpio* L. n = 3, and *Rutilus rutilus* L. n = 3, pooled for analyses) which represent the most abundant aquatic prey. Additionally, muscle samples of pigeons were collected on in 2010 (n = 3) and 2011 (n = 3) from local hunting society within 2 km of the study site where pigeons forage. No other potential terrestrial prey sources were observed in the study area. Samples for SIA were oven dried (60°C for 48 h) and analyzed at the Cornell Isotope Laboratory (COIL, Ithaca, NY).

### (c) Mixing Models

A Bayesian mixing model (Stable Isotope Analysis in R, SIAR) [19] was used to estimate the contribution of each prey (fish, crayfish and pigeon) to the diet of each individual European catfish (function *siarsolomcmcv4*, 500000 iterations) since it accounts for potential variability in the stable isotope values of consumers, prey and trophic fractionation [19]. Since no specific trophic fractionation values exist for European catfish, we followed a conservative approach [18] that consisted in using commonly reported fractionation factors with error estimates (1 ‰ (±1 SD) and 3.4 ‰ (±1 SD) for δ^13^C and δ^15^N, respectively) [16], [20]. For each individual, the lower and upper 50, 75 and 95% Bayesian credibility intervals of the contribution of each prey were predicted by the mixing models. Potential effects of individual body size, sampling date and sampled tissue on the mean predicted contribution of pigeon to the diet of each individual were tested using Spearman correlations and Kruskal-Wallis test, respectively. All statistical analyses were performed using R [21].

## Results

### (a) Behavioral Observations

During the 24 surveys conducted in an urbanized stretch of the Tarn River (France), between 1 and 9 European catfish (mean 3.9±2.1 SD, estimated body size range: 900–1500 mm) were observed swimming nearby a small island where pigeons regrouped for drinking and cleaning (Figure 1). Fifty-four beaching behaviors with partial and mostly complete stranding were observed and filmed (Movie S1), among which 28% (n = 15) were successful, i.e. the land birds were captured on land, returned to the river and swallowed (Figure 1 and Movie S1). On one occasion, complete stranding was observed but the attack was unsuccessful in capturing the pigeon. In approximately 40% of all observations, European catfish had more than half of their body outside of the water. The beaching behavior was quick, lasting from less than one second to no more than 4 seconds. The attacks were systematically triggered by active pigeons. Indeed, motionless pigeons, even very closed to the European catfish, were never attacked. Before the attack, European catfish were observed to exhibit erected upper jaw barbels on the upper jaw when they approach pigeons, suggesting that water vibrations, rather than visual cues, were used to detect and attack the prey.

### (b) Stable Isotope Analyses

Stable isotope analyses of European catfish revealed a high level of trophic niche variability among individuals, notably for δ^13^C that ranged from −26.6 ‰ to −22.8 ‰ while the variability in δ^15^N was somewhat lower (12.1 ‰ to 14.7 ‰, Figure 2). As fish and crayfish were ^13^C-depleted compared to pigeons (Figure 2 and Table S1), this indicated varying importance of aquatic (i.e. fish and crayfish) and terrestrial (i.e. pigeons) prey in the diet of the sampled individuals. Specifically, mixing models predicted a highly variable dietary contribution of pigeons among individuals, with 95% Bayesian credibility intervals ranging from 0–51% to 12–78% (Figure 3). While the dietary contribution of crayfish was relatively similar among individuals, the dietary contribution of pigeons increased as the dietary contribution of fish decreased (Figure 3). No significant effects of sampling date and sampled tissue on the mean predicted dietary contribution of pigeons were observed (Spearman correlation, P = 0.203 and Kruskal-Wallis test, P = 0.317, n = 14, respectively). However, the estimated body size range of European catfish observed hunting for pigeons (range: 900–1500 mm) tended to somewhat smaller that the estimated body size range of the individuals sampled in the study area (range: 900–2000 mm), suggesting a potential effect of individual body size on the display of this novel beaching behavior. This was partially confirmed by the existence of a negative but marginally significant relationship between individual body size and the mean predicted dietary contribution of pigeons (Spearman correlation, r = −0.60, P = 0.065, n = 14).

## Discussion

Although the consumption of terrestrial prey by aquatic predators is a ubiquitous phenomenon [4], [22], we document here a novel behavior displayed by an alien freshwater predator that was not, to the best of our knowledge, reported in its native range. This behavior allowed the capture of birds on land through intentional crossing of the water-land interface (beaching). Introduced species can display ecological and evolutionary adaptations in their new environment, and the occurrence of new behaviors can increase invasive species success [23]. Here, this new hunting strategy leads to a high level of trophic variability among individuals. Theories predict that behavioral and trophic specialization can have strong ecological and evolutionary consequences on intraspecific competition and individual fitness [8], which might subsequently affect the invasive success of the population. However, since European catfish were not monitored at the individual level in the present study, the potential correlation between success rate of attacks, dietary contribution of pigeons and individuals fitness were not tested. Therefore, it would be of great interest to determine the individual ecological consequences of this hunting behavior in an introduced population.

Understanding the ecological causes triggering the occurrence and maintenance of this unusual predation behavior is important, but it remains unknown at this stage. The emergence of trophic specialization in wild populations can be triggered by an increased intraspecific competition caused by an increased population density and/or a decreased in prey availability [10], [24]. European catfish have recently widely expanded its non-native distributional range through multiple introductions and colonization [11], [25], and although temporal patterns of the fish community in the study area are unknown, a potential increased density of European catfish and/or a potential decreased in prey fish might have caused this behavior to occur. This hypothesis might be explained by the existence of a negative and marginally significant relationship between individual body size and the dietary contribution of pigeons, assuming that smaller individuals are less competitive than larger individuals to prey upon prey fish. Alternatively, the risk of being stranded on the riverbank and the energy cost of attacking a pigeon on land might be lower for smaller individuals than for large-bodied specimen (i.e. >1500 mm). In this case, the costs associated with displaying this new beaching behavior (e.g. learning, risk of being stranded) might be counterbalanced by high energy returns provided by the consumption of the new prey, as observed elsewhere [4]. Therefore, land birds certainly represent a new ecological opportunity [10] that increases the diversity of trophic resources available to the introduced predator and that could drive individual specialization, as observed in other populations of predators foraging on allochtonous prey (e.g. [26]).

In conclusion, these findings suggest that this new predation behavior might represent an extreme example of the ability of introduced species to adapt to a new environment that could have unexpected implications for consumer-resources dynamics and ecosystem functioning [27], [28] that deserve further investigations.

## Supporting Information

Table S1 Stable isotope values of the three potential prey sources (fish, crayfish and pigeon) used in the mixing models. Reported values are the number of sampled individuals (n) and mean (± SD) δ^13^C and δ^15^N (in ‰).
(DOC)

Movie S1 Movie showing European catfish displaying beaching behavior to capture land birds, with two successful and two unsuccessful attacks.
(WMV)
