# Supplementary material for: “Freshwater Killer Whales”: Beaching Behavior of an Alien Fish to Hunt Land Birds
Source: PLoS One. 2012 Dec 5;7(12):e50840. doi: 10.1371/journal.pone.0050840 (PMC3515492; doi:10.1371/journal.pone.0050840)
Supplement: Table S1 — Stable isotope values of the three potential prey sources (fish, crayfish and pigeon) used in the mixing models. Reported values are the number of sampled individuals (n) and mean (± SD) δ13C and δ15N (in ‰). (DOC) [file pone.0050840.s001.doc]

**Supplementary Material S2**: Stable isotope values of the three potential prey sources (fish, crayfish and pigeon) used in the mixing models. Reported values are the number of sampled individuals (n) and mean (± SD) δ^13^C and δ^15^N (in ‰).

| **Origin** | **Name** | **Scientific name** | **n** | **δ^13^C** | **δ^15^N** |
| --- | --- | --- | --- | --- | --- |
| Aquatic | Fish* | *Blicca bjoerkna* | 9 | -26.26 (± 0.82) | 10.51 (± 0.95) |
|  |  | *Rutilus rutilus* |  |  |  |
|  |  | *Cyprinus carpio* |  |  |  |
|  | Crayfish | *Orconectes limosus* | 3 | -25.81 (± 0.05) | 9.21 (± 0.14) |
| Terrestrial | Pigeon | *Columbia livia* | 6 | -24.69 (± 7.09) | 1.23 (± 0.68) |

* the three species were pooled in the mixing models.
